# Evaluating the coverage and quality of nutrition programs via a bottom‐up approach: A secondary analysis of real‐time data from an end‐user monitoring system in Ethiopia

**DOI:** 10.1111/mcn.13360

**Published:** 2022-04-12

**Authors:** Anne‐Sophie Donzé, Abiy Tefera, Kaleab Baye, Stéphane Arnaud, Stanley Chitekwe, Arnaud Laillou

**Affiliations:** ^1^ Nutrition Section, UNICEF Ethiopia Addis Ababa Ethiopia; ^2^ Center for Food Science and Nutrition Addis Ababa University Addis Ababa Ethiopia; ^3^ Supply Chain Section, UNICEF Ethiopia Addis Ababa Ethiopia

**Keywords:** end‐user monitoring, Ethiopia, nutrition counselling, nutrition services, quality

## Abstract

Over the last two decades, great efforts and investments have been made in Ethiopia to ensure that all children have equal access to nutrition services in health facilities. While quality health systems are a prerequisite for quality nutrition services, little attention has been given to the evaluation of the supply and delivery services. The purpose of the study was to evaluate the coverage and quality of the nutrition‐specific interventions delivered through the health system. Using an end‐user monitoring (EUM) system, we monitored the delivery of nutrition‐specific interventions in 500 districts, having 2514 health facilities distributed throughout Ethiopia. Data were collected through third‐party monitors between August 2020 and 2021. Roughly 90% of health facilities were performing severe acute malnutrition management in line with the national guideline/protocol, and 2/3 of the assessed facilities were delivering iron and folic acid, vitamin A supplementation and deworming. A third of the messages on AMIYCN were retained by beneficiaries. Warehouse conditions were good in 64.3% of the facilities, but only 22% had good recording practices and about half had problems related to the quality and availability of nutrition supplies. Most beneficiaries were satisfied with the nutrition supplies and service delivered at the health facility level. This study also suggests the relevance of an EUM system to assess the quality of nutrition service delivery and its related supply management, as well as to improve the implementation of nutrition interventions as a decision‐making tool.

## Key messages

- The main bottleneck identified in supply management was the recording system, while roughly 65% of warehouses are in good condition to store nutrition supplies at the health facility level.
- Roughly 90% of health facilities were performing severe acute malnutrition management in line with the national guideline, while 2/3 of the health facilities are delivering iron and folic acid, vitamin A supplementation and deworming.
- The knowledge and practices of the HW were above average and only a third of the messages delivered were retained by beneficiaries
- Although messages are being well delivered once counselling is implemented, many opportunities (contact points) are missed to deliver nutrition counselling.

## Graphical abstract

The purpose of the study was to evaluate the coverage and quality of the nutrition‐specific interventions delivered through the health system. Using an end‐user monitoring (EUM) system, we monitored the delivery of nutrition‐specific interventions in 500 districts, having 2514 health facilities distributed throughout Ethiopia. This study suggests the relevance of an EUM system to assess the quality of nutrition service delivery and its related supply management, as well as to improve the implementation of nutrition interventions as a decision‐making tool.

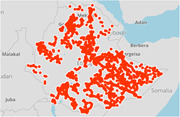

## 1 INTRODUCTION

Accelerated and expanded efforts will be required to meet Sustainable Development Goal 2 and its target of ending all forms of malnutrition. Despite progress, 36.8% and 7.2% of the roughly 15 million children under five in Ethiopia are suffering from stunting and wasting, respectively (Ethiopian Public Health Institute [EPHI] & ICF, 2019). Moreover, out of the 67 deaths per 1000 live births among children under five, 45% of the under‐five mortality is associated with undernutrition (Central Statistical Agency [CSA] [Ethiopia] & ICF, 2016; World Health Organization, 2018). Many of the nutrition‐specific interventions, such as severe acute malnutrition (SAM) treatment and vitamin A supplementation (VAS) for children under five, iron and folic acid (IFA) supplementation for pregnant women, deworming with albendazole for children aged 1–5 years, and Maternal, Infant and Young Child Nutrition (AMIYCN) counselling for pregnant and lactating women and caregivers of children under two, are delivered through the health system, but global nutrition movements to scale‐up effective nutrition interventions and the visions to achieve universal health coverage have not been well connected.

Health system strengthening provides many opportunities to improve nutrition services, such as ensuring that IFA supplements are given during all antenatal care (ANC) visits and exclusive breastfeeding is promoted during the immunization of infants. An analysis reported by the World Bank showed that scaling up the key nutrition‐specific interventions to 90% coverage along with expected improvements in the underlying determinants of stunting are estimated to lead to about a 19.5% decline in the number of stunted children by 2025 in 37 high‐burden countries, including Ethiopia (Shekar et al., 2016). Similarly, the 2017 Global Nutrition report estimated that every US$1 spent by donors on basic nutrition programs will lead to a US$ 16 return to the local economy (Development Initiatives, 2017). In 2019, the World Health Assembly urged for a stronger focus on nutrition within health facilities to save lives and focus on addressing the Essential Nutrition Actions (World Health Organization, 2019).

Over the last two decades, great efforts have been made in Ethiopia to ensure that all children have equal access to nutrition services delivered through health facilities and community‐based programs. The National Health Extension Programme (HEP) has played a critical role in increasing the coverage of services in remote and rural Ethiopia (Medhanyie et al., 2012). However, gaps in coverage and inequalities in access to health care are impeding further progress needed to maximize the impact of nutrition interventions on child health and well‐being (Baye, 2019). The limited regular household visits and growth monitoring of children by health extension workers (HEWs) as reported by households illustrates the inconsistency of the quality of the service delivered (Warren & Frongillo, 2017). Besides, key nutrition messages regarding breastfeeding and complementary feeding came from different sources carrying inconsistent counselling (Warren & Frongillo, 2017).

Improvement in the quality of health services requires close monitoring through real‐time data, but this has been identified as a critical gap in a recent paper that evaluated coverage of nutrition interventions within health systems in low‐ and middle‐income countries (LMICs) (Heidkamp et al., 2020). Besides, most of the nutrition interventions recommended by the lancet series to improve maternal and child undernutrition need cross‐cutting strategies, such as effective supplies delivery and monitoring, as well as accountability (Keats et al., 2021). The assessment of the quality of health services has been mostly focused on the coverage of interventions, but little effort has been put into monitoring the quality of the services delivered. Unfortunately, a limited system exists in most countries to assess the last mile distribution of those commodities to the beneficiaries and ensure their satisfaction.

**Figure 1:**
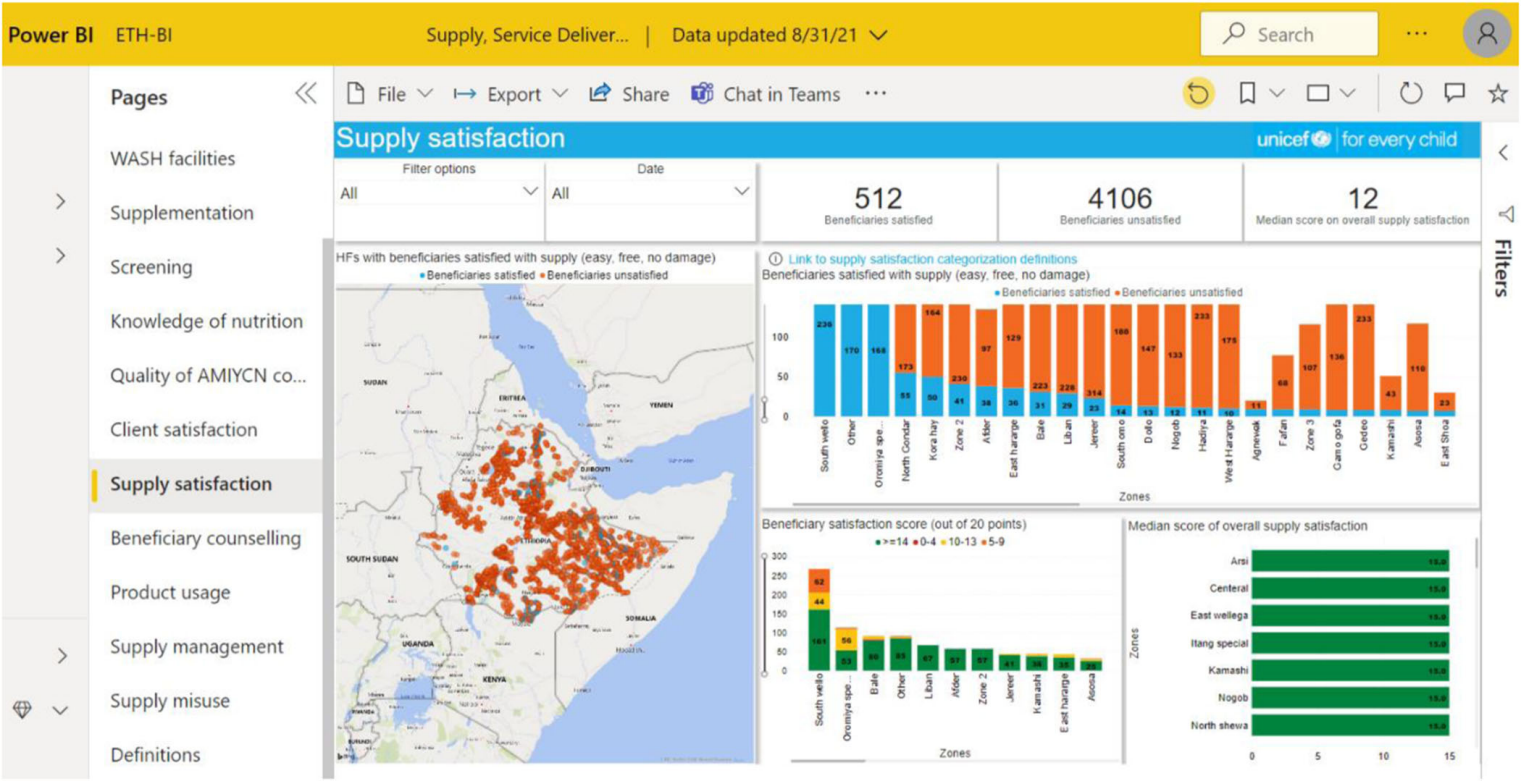
Screenshot of the dashboard (31/08/2021) generated from open‐data kit, with an example on supply satisfaction

Within this context, UNICEF has developed an end‐user monitoring (EUM) system, gathering data on supply management, and nutrition services coverage and quality at health facility level via third‐party monitors (TPM). The quality of nutrition services is evaluated by assessing the nutrition supply management, the knowledge of HW, the interaction between HW and beneficiaries and the satisfaction of beneficiaries with the nutrition service and supply delivered. UNICEF has created a dashboard (see Figure 1) to monitor the management of supplies and nutrition service delivery at the health facility level.

Therefore, the aim of the study was to evaluate the coverage and quality of the nutrition‐specific interventions and supplies delivered through the national health system in Ethiopia. To do so, this study is the first to use the monitoring data gathered through this EUM system, which has been implemented in over 500 districts of Ethiopia.

## 2 METHODS

### 2.1 Study setting

Ethiopia is the second‐most populous country in Sub‐Saharan Africa, with 117.8 million inhabitants and an area of 1.1 million square kilometres. Ethiopia is structured into nine regional states, further decentralized to zonal and district levels. The Ethiopian health system has three levels: at the primary‐care level, a primary care unit consists of a primary hospital, a health centre and a health post. The secondary is composed of general hospitals, which serve as referral centres for primary hospitals, while the tertiary‐care level represents specialized hospitals, serving as referrals from general hospitals (Wang et al., 2016). There are roughly 22,000 health facilities across the country and to ensure the delivery of essential health services to most of the Ethiopian population who lives in rural areas, an HEP was established (The Federal Democratic Republic of Ethiopia Ministry of Health, 2019; Wang et al., 2016). It is a community‐based program, providing primary‐care level services, including nutrition services, at the household level, where two HEWs are deployed for each health post serving 3000 to 5000 people.

The Ethiopian health system has integrated routine nutrition services that are routinely delivered through the health system: SAM treatment and VAS for children under five, IFA supplementation for pregnant women, deworming with albendazole for children aged 1–5 years and AMYICN counselling for pregnant and lactating women and caregivers of children under two. The DHIS2, the Health Information System of the FMoH, collects nutrition data for each of these interventions at the district level. To deliver the abovementioned nutrition services, adequate nutrition supplies are needed: RUTF (ready‐to‐use therapeutic food) for SAM treatment, vitamin A and IFA supplements and albendazole against soil‐transmitted helminths.

Therefore, a crucial element of nutrition service delivery is supply management at the facility level. HEWs are responsible for supply monitoring, comprising the following recording system. Upon receipt of nutrition supplies from the district level, the health facility receives so‐called issuing vouchers from the district and gives receiving vouchers in return. Bin cards (also referred to as inventory ledger) are under the form of a table indicating quantities received, issues, lost and remaining balance for each supply. They are used for supplies stock monitoring and for supply refill requests.

### 2.2 Monitoring design

The present study used data collected through an EUM system, developed by UNICEF. This EUM system is an Information and Communication Technology tool using a real‐time monitoring system. The EUM is built on an open‐source technology called open‐data kit (ODK), which is free and sets tools that can assist in creating, authoring and managing mobile data collection processes. Upon a visit to a health facility, the data was directly collected into the mobile‐based ODK app. Once the questionnaire is completed, the data would automatically be uploaded into the EUM database.

TPM—roughly 30 national officers with qualification in public health or nutrition—were hired to support the EUM. TPM received the following training on data collection with the ODK embedded in a mobile application: between 17–20 August 2020, a 1‐day training programme was organized in two half‐day sessions for the TPM in all regions of Ethiopia. Moreover, 2 days of virtual training were organized from 31 March–1 April 2021 to refresh the TPM on nutrition‐specific services and data collection via the EUM system. The training was provided by UNICEF staff, including monitoring and evaluation, nutrition and IT specialists.

From August 2020 to August 2021, UNICEF through third‐party monitoring collected data, yielding 4618 data monitoring reports on health facilities. Every day, on average 13 health facilities are monitored by TPM using the EUM tool. During this 1‐year data collection period, 2413 health facilities were assessed, where 6.9%, 38.4% and 54.7% of the data monitoring reports (*n* = 4618) were administered in hospitals, health centres and health posts, respectively. TPM randomly selected health facilities visited within the geographical area they had been requested to cover. As for the selection of the HW and beneficiaries for interview, it is simply based on their presence on the day of the health facility monitoring visit. Each TPM and roughly a fifth of the health facilities were visited more than once for assessment. There are two reasons for third‐party monitoring to assess more than once the same health facility: (1) the third party can submit a partially completed EUM questionnaire to the database, for example, due to the absence of an AMIYCN counselling session on the day of data collection, and (2) if any corrective action was taken by the third party, for example, a training was given on supply management following a health facility visit reporting low performance on that aspect.

**Figure 2:**
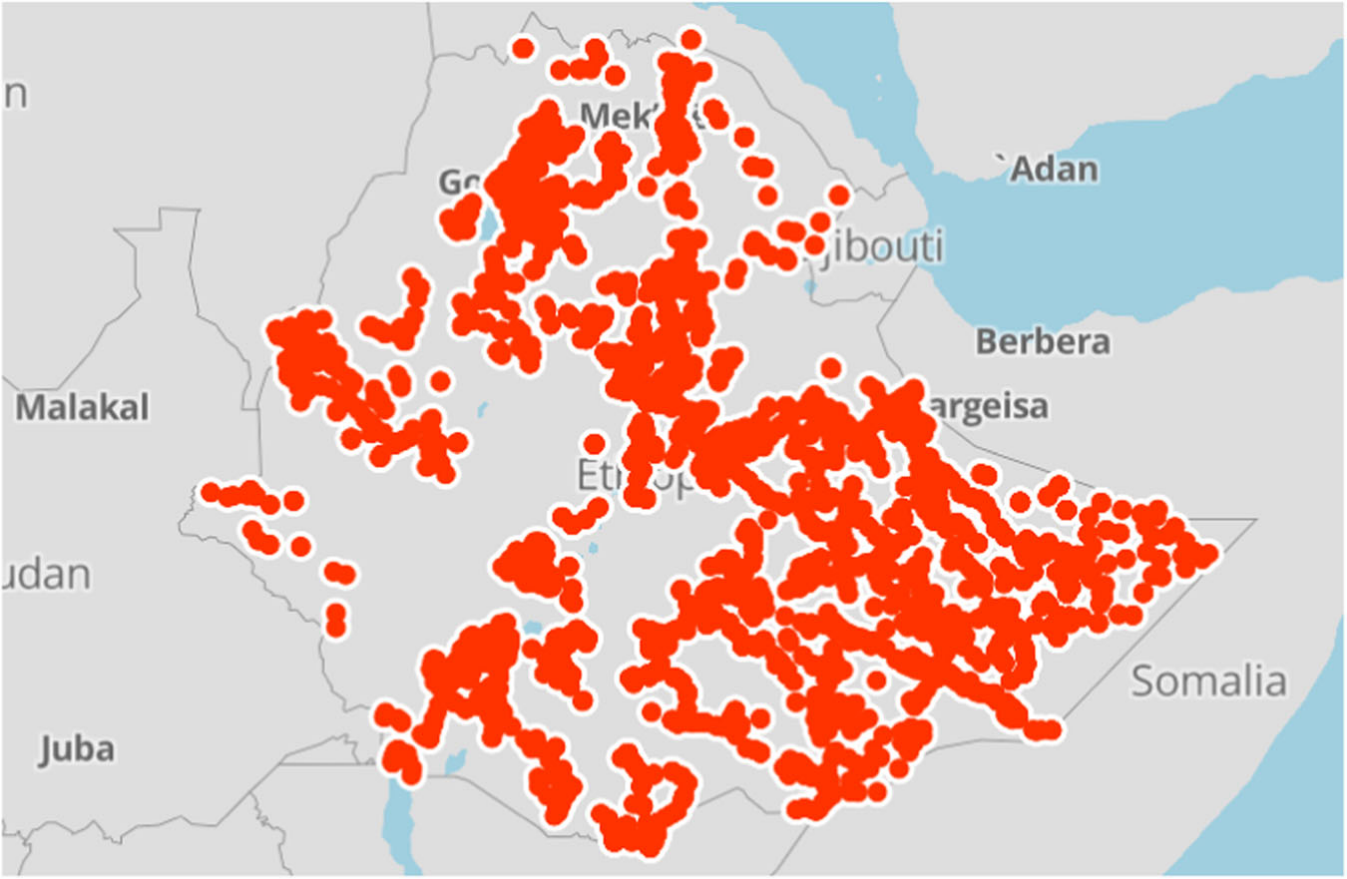
GPS coordinates of health facilities visited by the third party for EUM data collection

TPM conducted interviews in all regions of the country: Oromia, Amhara, SNNPR/Sidama, Somali, Tigray, Afar, Gambella and Benishangul‐Gumuz, as well as in two administrative towns of Ethiopia (Harari and Dire Dawa). This monitoring exercise was performed in 520 districts across the country. Figure 2 illustrates the geographical distribution of the 2514 randomly selected health facilities for monitoring visits during the 1‐year period of data collection. With roughly 10% of the health facilities in Ethiopia having been monitored through the EUM system and the spread of the visits across the country, the data used for this study was assumed to likely be a representative sample for evaluating regional and national coverage and quality of nutrition services.

### 2.3 Data collection

During a monitoring visit to a health facility, TPM directly would answer the questionnaire in the ODK mobile app linked to the EUM system. To complete this questionnaire, the TPM collected data from registers, interviews with both health workers and beneficiaries and observation of service delivery. The questionnaire assessed the coverage and quality of nutrition service delivery at the health facility level. The EUM questionnaire consists of different modules: supply management, service delivery and beneficiary satisfaction. Following a health facility monitoring visit, TPM could submit questionnaires without answering all modules.

#### 2.3.1 Coverage

TPM evaluated the coverage of key nutrition‐specific interventions (VAS, albendazole, screening, SAM treatment, IFA for pregnant women and nutrition counselling) at the health facility level. TPM based their service delivery assessment on interviews with HWs and registers, such as OTP cards and bin cards for nutrition supplies. Health facilities were considered as delivering the management of SAM and supplementation when both admissions and discharge of children under five are recorded, and when iron‐folic acid has been provided to pregnant women, vitamin A to children aged 6–59 months and albendazole to children 24–59 months, respectively.

#### 2.3.2 Quality

TPM assessed the quality of nutrition‐specific interventions delivered, by evaluating the nutrition supply management, the knowledge of HW, the interaction between HW and beneficiaries and the satisfaction of beneficiaries on the nutrition service delivered.

Data on supply management were collected from the storage room dedicated in health facilities and on damage, expiration, losses, misuse and any stock out of supplies. TPM based their supply management evaluation on interviews with HWs and registers. A score (out of 8) was computed to assess the quality of supply management through the following 8 indicators: (i) the warehouse is in good condition (1 pt), (ii) inventory ledger or bin card for products (1 pt), (iii) inventory record up‐to‐date and accurate (1 pt), (iv) no product shortage (1 pt), (v) no products damaged (1 pt), (vi) no product expired (1 pt), (vii) no products damaged (1 pt) and (viii) the facility have issuing voucher for its consignees (1 pt). If the supply management score equals or is greater than 6, the health facility is considered to have good supply management. To further assess the supply management score, these eight indicators were grouped in three main categories, that is storage conditions (i), quality and availability of products (iv, v, vi, vii) and recording system (ii, iii, viii). Warehouse conditions, quality, availability of products and recording system will be considered as good when all their respective indicators are fulfilled. A health facility scoring ≥6 was considered good at supply management.

Another aspect of the EUM system to evaluate the quality of nutrition service delivery is the assessment of HW knowledge. TPM directly interviewed the HW, testing their capacity to provide AMIYCN counselling and knowledge of who each nutrition supply is for and for which nutrition service it is provided. The score (out of 20) is calculated as follows: there are 28 facts that the HW must know for each beneficiary's age group (nutrition counselling for pregnant women, caregivers of infants aged 0–6 months, young children aged 6–23 months and of children aged 24–59 months, as well as for adolescents). Each fact correctly known by the HW is equivalent to 1 pt, and the total score was then computed by dividing the sum by 1.4.

To further evaluate the quality of nutrition service delivery, the interaction between the HW and the beneficiaries was determined by TPM, by observing AMIYCN counselling sessions. The score of this interaction was assessed through eight indicators (20pts): (i) communicate based on the client's knowledge, cultural values and beliefs (1pt), (ii) listen carefully and actively to the beneficiary's concern (1pt), (iii) ask an open‐ended question (1pt), (iv) praise beneficiary's good practices (1pt), (v) suggest actions that are acceptable, affordable and feasible (2pts), (vi) discuss on the follow‐up appointment (2pts), (vii) give opportunity for question (1pt) and (viii) use counselling card (1pt). Hence 10 more points were attributed according to the number of messages passed by the HW and that beneficiaries were able to remember depending on the required counselling topic (either for pregnant women, for mothers with children aged 0–6 months, or 6–23 months, or 24–59 months, or for adolescents). This information was collected by the TPM by asking the beneficiary which counselling messages they had been provided with from the HW.

The last aspect of the evaluation of the quality of nutrition service delivery consisted in the satisfaction of the beneficiaries. TPM evaluated the beneficiaries' satisfaction by directly interviewing them at the health facility and using a scale from 0pt (*very dissatisfied*), 1pt (*dissatisfied*), 2pts (*neither*), 4pts (*satisfied*) to 5pts (*fully satisfied*). First, the beneficiary was asked for their overall service delivery satisfaction, as follows: (i) waiting time, (ii) privacy space for consultation, (iii) use of information and education materials and (iv) overall interaction with the HW. If their score was equal to or higher than 12, the beneficiaries were considered satisfied with the service delivery.

Second, a score (out of 15) on the satisfaction of the nutrition supply provided to beneficiaries was assessed using the same scale (0–5pts), consisting of the following aspects: (i) products were easy and safe to use, (ii) products were given in adequate quantities and (iii) products were effective in improving the nutritional status of the beneficiary.

### 2.4 Statistical analyses

While a dashboard was developed by UNICEF to have a user‐friendly data visualization of the database from data collected in the field for monitoring purposes, the present study is a secondary data analysis of the EUM system database. Data management, including quality checks, and analysis were performed with Microsoft Excel® and SPSS® vs20. The data were only accessible to UNICEF staff. The authors had hence the permission to extract the data. The raw data does not include identifiers therefore there was no institutional ethics approval needed to analyse the data. The data storage is hosted by Ona Systems® and can be shared by UNICEF staff with access to the data. We have then assessed the normal distribution of the knowledge, observation and beneficiary scores (over 20) through the test of Shapiro–Wilk. Wilcoxon's signed‐rank tests and *χ* ^2^ tests—with continuous and categorical variables, respectively—were conducted to compare means of nutrition service, supply management indicators and beneficiary satisfaction between 2020 and 2021.

## 3 RESULTS

### 3.1 Coverage of nutrition services

The proportion of health facilities delivering nutrition services at both national and regional levels is depicted in Table 1. Of the 2413 health facilities assessed for key nutrition‐specific services, 88% were performing admission of SAM children in line with the national guideline/protocol, 3/4 were delivering vitamin A (76%) and albendazole (77%). IFA was delivered to pregnant women in 79% of the health facilities. While more than 75% of health facilities were fully delivering either VAS, IFA or Albendazole, 2/3 of health facilities assessed were delivering all three supplementation interventions.

**Table 1:** Coverage of health facilities delivering nutrition services at the national and regional levels

		%			
	*N*	Treatment of SAM	Vitamin A supplementation	Iron‐folic acid supplementation	Albendazole (deworming)
National	2514	88 (87, 90)	76 (74, 77)	79 (78, 91)	77 (75, 78)
Afar	221	94 (91, 97)	97 (95, 99)	98 (96, 100)	96 (93, 99)
Amhara	531	84 (81, 87)	84 (81, 87)	95 (94, 97)	85 (81, 88)
Benishangul‐Gumuz	64	81 (71, 91)	83 (73, 92)	84 (75, 94)	84 (75, 94)
Gambella	15	87 (67, 107)	67 (40, 94)	93 (79, 108)	87 (67, 108)
Oromia	429	77 (73, 81)	79 (75, 83)	93 (91, 96)	79 (75, 83)
SNNP/Sidama	281	90 (87, 94)	86 (82, 90)	88 (84, 92)	87 (83, 91)
Somali	955	95 (93, 96)	61 (58, 64)	56 (53, 60)	63 (60, 66)
Tigray	9	67 (28, 105)	78 (44, 112)	78 (44, 112)	89 (63, 115)

*Note*: Data are represented in percentage values (95% CI: lower bound, upper bound). Data collected in Dire Dawa and Harari administrative towns are not reported individually but are included in the national data.

Abbreviations: CI, confidence interval; SAM, severe acute malnutrition.

**Figure 3:**
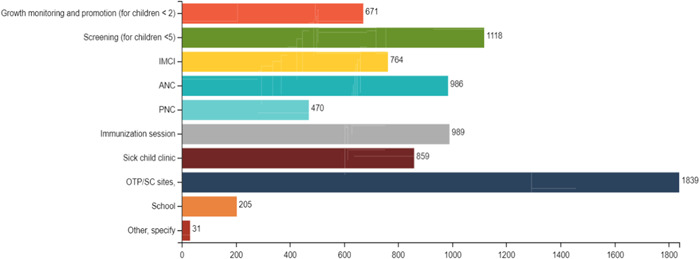
Contacts points used by the health staff to provide nutrition counselling in absolute numbers. ANC, antenatal care; IMCI, integrated management of childhood illness; OTP/SC, outpatient therapeutic programme/stabilization centres for wasting treatment; PNC, postnatal care

For nutrition counselling, the main contacts acknowledged by the health staff are OTP (outpatient therapeutic programme)/SC (stabilization centre) sites (23.2%), screening for children under 5 years of age (14.1%), immunization sessions and ANC (around 12.5% both), while growth monitoring and promotion (GMP) only represented 8.4% of the contact points. Figure 3 depicts the relevance of all the contact points used by health workers assessed for the coverage of nutrition counselling.

The coverage of SAM management according to the national guidelines and supplementation among health facilities varied across regions. Somali, Afar and SNNP/Sidama regions show the highest coverage (≥90%), followed by Amhara, Benishangul‐Gumuz and Gambella with coverage lying between 80% and 90%. Oromia and Tigray regions are characterized by coverage of SAM treatment lower than 80%. As for the supplementation services, Afar is the region with the highest proportion of health facilities delivering VAS (97%), IFA (98%) and deworming (96%). Somali Region shows the lowest coverage for VAS (61%), IFA (56%) and deworming (63%).

### 3.2 Quality of nutrition services

Table 2 depicts the different scores assessing the quality of nutrition services delivered in health facilities at national and regional levels. Health facilities scored a median of 6 over 8 points during the supply management assessment, with half of the facilities being considered as having good supply management. Of the 2374 health facilities assessed via the module on supply management, 64.3% had good warehouse conditions, 21.8% showed a good recording system and 50.7% did not have any issues regarding the quality and availability of nutrition supplies. Amhara, Gambella and Somali regions were found with the same score as at national level, while Afar, Benishangul‐Gumuz, Oromia, SNNP/Sidama and Tigray scored lower than the national level.

**Table 2:** Different scores assess the quality of nutrition services delivered in health facilities at the national and regional levels

	Supply mgmt. score^a^		HW knowledge score^b^		Interaction score^b^		Supply satisfaction score^c^		Service satisfaction score^b^	
	Median	*N*	Median	*N*	Median	*N*	Median	*N*	Median	*N*
National	6.0 (2)	2374	14.0 (6)	2514	16.0 (6)	2514	12.0 (2)	726	16.0 (4)	1202
Afar	5.0 (2)	220	14.0 (4)	221	15.3 (5)	221	12.0 (0)	74	9.0 (3)	106
Amhara	6.0 (2)	530	15.0 (4)	531	13.7 (8)	531	12.0 (5)	120	14.0 (4)	311
Ben.‐Gum.	5.0 (2)	138	13.0 (6)	64	14.4 (7)	64	14.0 (3)	39	15.0 (5)	41
Gambella	6.0 (4)	17	11.0 (7)	15	10.7 (8)	15	12.0 (5)	2	13.0 (8)	11
Oromia	5.0 (2)	324	13.0 (5)	429	14.3 (5)	429	13.0 (1)	103	14.0 (4)	138
SNNP	4.0 (3)	177	14.0 (7)	281	15.0 (3)	281	12.0 (2)	58	16.0 (2)	81
Somali	6.0 (2)	946	16.0 (7)	955	18.7 (4)	955	12.0 (1)	319	16.0 (1)	511
Tigray	5.5 (2)	12	14.0 (9)	9	13.0 (6)	9	15.0 (3)	4	N/A	1

*Note*: Data are reported in median (IQR). SNNP includes data from the Sidama Region. Data collected in Dire Dawa and Harari administrative towns are not reported individually but are included in the national data.

Abbreviations: Ben.‐Gum., Benishangul‐Gumuz; Mgmt., management.

^a^ Score is out of 8pts.

^b^ Score is out of 20pts.

^c^ Score is out of 15pts.

During the assessment period of the nutrition‐specific services in health facilities, 2374 evaluations were performed on the management of supply and even though products that were damaged (6.1%), expired (12.2%), lost (1.6%), or misused (4.6%) were limited in the 2,413 health facilities assessed, 42.6% have experienced stock‐out. The main stockout was for RUTF (22.7%), while vitamin A and deworming represented around 13% each of the stockouts, and IFA 8.2%.

The EUM has given a median score of 14.0 over 20 points upon assessment of the knowledge of HW. The percentage of appropriate topics explained by the HEWs to beneficiaries varied across counselling types: on average 6 messages are provided out of 8 for mothers with children 0–6 months, on average 2 messages are provided out of 4 for adolescents, on average 3 messages are provided out of 6 for pregnant women, on average 1 message are provided out of 3 for mothers with children aged 24–59 months and on average 2 messages are provided out of 6 for mothers with children aged 6–23 months. The regions with a score of HW knowledge equal or higher than the national score are Afar, Amhara, SNNP/Sidama, Somali and Tigray. The knowledge of HW is Benishangul‐Gumuz, Gambella and Oromia was reported to be lower than the national score.

The interaction between the health staff and beneficiaries was given a median score of 16 out of 20 including the nutrition counselling messages retention of beneficiaries. Messages for adolescents seem to be the less well retained by the beneficiaries as only 26.3% recall at least half of the message (*n* = 161), while 94.1% of the pregnant women (*n* = 823), 87.9% of the mother with child 0–6 months (*n* = 601), 86.6% of the mother with child 6–24 months (*n* = 1137) recall at least half of the messages. According to beneficiaries, the two main suggested areas of improvement for HEWs during counselling are to make the counselling more practical and to give more time for counselling services.

**Figure 4:**
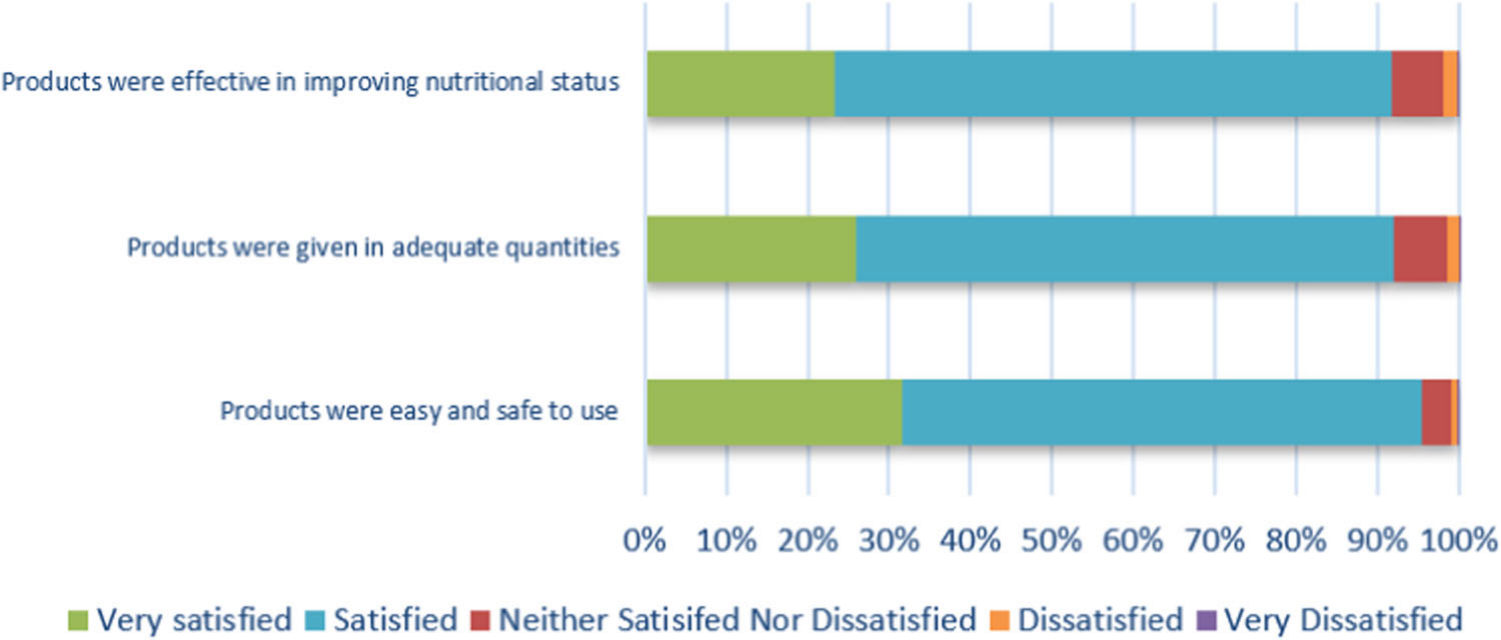
Satisfaction rate of the beneficiaries on nutrition supply received after health facility visit (*n* = 1311)

The satisfaction of beneficiaries concerning the supplies they have received at the health facility was given a median score of 12 out of 15 and is illustrated in Figure 4. All regions indicated a score equal to or higher than the national score. As for the level of satisfaction with the overall service delivery at the health facility level, it was characterized by a good score of 16 out of 20. Only the satisfaction score in Afar was lower than 12 indicating that the beneficiaries were not satisfied with the service delivery.

## 4 DISCUSSION

The EUM in this study was intended to be used to assess service readiness and nutrition service provision across Ethiopia and results indicated a relatively good coverage of service delivery. Nearly 88.5% of health facilities were performing admission of SAM children in line with the national guideline/protocol, and 2/3 of health facilities assessed were fully delivering the three supplementation interventions. Yet, according to January‐June 2021 data from UNICEF and the Ethiopian FMoH, the coverage of supplementation varied across interventions: 72% for children aged 6–59 months with VAS, 65% for children aged 24–59 months with albendazole for deworming and only 34% for pregnant women with IFA. The high coverage of SAM treatment is probably linked to the launch in 2019 of revised national guidelines for community‐based management of acute malnutrition, and consequent training across the country. As no major problem was identified concerning the management of supplementation supplies, the difference in coverage is suggested to lie in the other aspects of service delivery quality, such as HW skills and beneficiary's satisfaction. As two recent studies led in Ethiopia recently noted, IFA lower coverage might be due to suboptimal counselling and misconception about the beneficial and side effects of IFA (Fenta et al., 2020; Gebre et al., 2015). This highlights the need for enhanced community sensitization and counselling about the relevance of IFA during pregnancy.

This study has also identified the main bottleneck in supply management at the health facility level, 78.2% of health facilities showed low‐performing recording systems. This indicates that, to improve supply management for nutrition service delivery in Ethiopia, priority should be first given to the improvement of the recording system. Our analysis also emphasizes the relevance of beneficiary monitoring to ensure that it is reaching the targeted population and not being misused. Given the evidence of increased disease and economic burdens caused by poor diets (Candari et al., 2017), it is essential to reduce the cost of healthcare by enhancing the supply management as well. For example, reducing the shortage of or the non‐efficient use of nutrition supplies could be cost‐effective actions for nutrition services. The EUM system, by gathering information on last‐mile distribution, including beneficiaries' feedback, could contribute to improving supply chain efficiencies (USAID, 2013).

While this study suggests that at least a third of the messages were retained by beneficiaries, poor nutrition counselling due to high caseload, staff shortage and capacity gap has been previously reported (Fenta et al., 2020). Although this is encouraging, future strategies could emphasize the messages that were not retained to improve their delivery and the counselling skills of health workers. However, such redesign of training should be cognizant of the already overwhelming workload of HEWs, as well as other challenges like incentives (Baye & Hirvonen, 2020; Fenta et al., 2020). A study conducted in rural Ethiopia had similar results showing a knowledge gap between the HEWs and the trained mothers on AMIYCN, and suggests to improve the knowledge sharing effectiveness of the HEWs (Abebe et al., 2016). For example, the strengthening of health service delivery to ensure that IYCF messages are repeated multiple times to mothers/caregivers has been identified as a key approach to enhance AMIYCN programs (Lutter et al., 2013).

Maximizing underutilized contact points such as the Integrated Management of Newborn and Childhood Illnesses (IMNCI), GMP and Post‐natal care (PNC) could further increase reach and frequency of nutrition messaging. For example, 69.4 million growth monitoring episodes (roughly 5.5 visits per child per semester) were performed in 2020 in Ethiopia according to the health information system (DHIS2) of FMoH. Two recent studies—conducted in India and Peru—demonstrated that GMP, inter alia, as a routine health service to deliver nutrition counselling was an efficient channel to improve feeding practices (Bhandari et al., 2005; Penny et al., 2005). Repetitive exposure and tailored AMIYCN messages seems to be the key mechanisms of success using GMP as a contact point for nutrition counselling (Ashworth et al., 2008; Bégin et al., 2020). Therefore, we might have to maximize potential of GMP, target younger children and strengthen the nutrition counselling elements, combine growth monitoring with other health intervention channels such as immunization for the convenience of caregivers and ensure consistent message delivery (Ashworth et al., 2008).

The following limitations should be considered when interpreting our findings. We have assessed 11% of the functional health facilities in the country, which is a consequent coverage, but the scope of the service might not be representative of all the health facilities in the country and might have a bias since the ones assessed were the ones prioritized by UNICEF. However, given the wide distribution of our sample across many districts and zones, we are confident that our findings can be relevant to areas that were not covered by our assessments. Some of the responses may also be prone to social desirability bias. Moreover, the regions of Tigray and Gambella were underreported, and hence most likely not representative, which might have been due to the limited possibility of implementing EUM caused by insecurity.

## 5 CONCLUSIONS

The findings of this study highlighted the relatively good coverage and quality of nutrition‐specific interventions delivered through the Ethiopian health system and identified some of its bottlenecks. This is the first study to monitor the delivery of nutrition‐specific interventions using this EUM system. This innovative tool can effectively and timely inform programming by using a bottom‐up approach and filling the gap in real‐time data. Such a real‐time monitoring system can help improve large‐scale nutrition interventions by identifying where efforts should be prioritized. This could contribute to strengthening the resilience of the health system. Such an EUM system could also include multisectoral data, such as food security, health, as well as water, sanitation and hygiene to fully tackle child and maternal malnutrition, not in only in Ethiopia but also in context‐similar countries.

## AUTHOR CONTRIBUTIONS

Arnaud Laillou, Kaleab Baye and Stéphane Arnaud designed the scope of the paper. Anne‐Sophie Donzé, Abiy Tefera and Arnaud Laillou conducted the analysis and wrote the first draft of the paper. Arnaud Laillou and Stanley Chitekwe reviewed the manuscript. All authors read and approved the final manuscript.

## CONFLICTS OF INTEREST

The authors declare no conflicts of interest.

## Data Availability

The data that support the findings of this study are available from the corresponding author upon reasonable request.
